# Delirium and Cognitive Impairment as Predisposing Factors of COVID-19 Infection in Neuropsychiatric Patients: A Narrative Review

**DOI:** 10.3390/medicina57111244

**Published:** 2021-11-14

**Authors:** Michele Fabrazzo, Antonio Russo, Alessio Camerlengo, Claudia Tucci, Mario Luciano, Valeria De Santis, Francesco Perris, Francesco Catapano, Nicola Coppola

**Affiliations:** 1 Department of Psychiatry, University of Campania “Luigi Vanvitelli”, Largo Madonna delle Grazie 1, 80138 Naples, Italy; alessiocamerlengo90@gmail.com (A.C.); claudiatucci3@gmail.it (C.T.); mario.luciano@unicampania.it (M.L.); valedesa1984@libero.it (V.D.S.); francesco.perris@unicampania.it (F.P.); francesco.catapano@unicampania.it (F.C.); 2 Infectious Diseases Unit, Department of Mental Health and Public Medicine, University of Campania “Luigi Vanvitelli”, Via S. Pansini 5, 80131 Naples, Italy; antoniorusso.ar.ar@gmail.com (A.R.); nicola.coppola@unicampania.it (N.C.)

**Keywords:** SARS-CoV-2, COVID-19, delirium, cognitive impairment, psychiatric history, mental health

## Abstract

SARS-CoV-2 neuroinvasive and neurotropic abilities may underlie delirium onset and neuropsychiatric outcomes. Only a limited number of studies have addressed the potential effect of SARS-CoV-2 infection on mental health so far. Most studies mainly reported the acute onset of mixed neuropsychiatric conditions in patients infected with SARS-CoV-2, characterized by agitated behavior, altered level of consciousness, and disorganized thinking, regardless of psychological or socioeconomic triggering factors. The present narrative review aims to analyze and discuss the mechanisms underlying the neuroinvasive/neurotropic properties of SARS-CoV-2 and the subsequent mental complications. Delirium appeared as a clinical manifestation of SARS-CoV-2 brain infection in some patients, without systemic or multiple organ failure symptoms. A small number of studies demonstrated that neuropsychiatric symptoms associated with COVID-19, initially presenting as a confused state, may subsequently evolve in a way that is consistent with the patients’ neuropsychiatric history. A literature analysis on this topic prevalently showed case reports and case series of patients presenting delirium or delirium-like symptoms as the main outburst of COVID-19, plus a cognitive impairment, from mild to severe, which pre-existed or was demonstrated during the acute phase or after infection. Dementia appeared as one of the most frequent predisposing factors to SARS-CoV-2 infection complicated with delirium. Instead, contrasting data emerged on the potential link between COVID-19 and delirium in patients with cognitive impairment and without a neuropsychiatric history. Therefore, clinicians should contemplate the possibility that COVID-19 appears as delirium followed by a psychiatric exacerbation, even without other systemic symptoms. In addition, cognitive impairment might act as a predisposing factor for COVID-19 in patients with delirium.

## 1. Introduction

Infection with severe acute respiratory syndrome coronavirus 2 (SARS-CoV-2), which caused coronavirus disease 2019 (COVID-19), is associated with a large spectrum of clinical manifestations [1]. COVID-19 may impact both mental and physical health, thus influencing the well-being of the general population worldwide [2,3]. Outcomes vary in severity, ranging from asymptomaticity to severe pneumonia and acute respiratory distress syndrome (ARDS) requiring admission to an intensive care unit (ICU) [4,5]. Furthermore, older adults with medical comorbidities such as diabetes, hypertension, and other cardiovascular diseases, are more exposed to severe complications of COVID-19 and have an increased risk of mortality [6].

COVID-19 typically manifests as an influenza-like respiratory illness in symptomatic individuals, with fever, cough, dyspnea, general malaise, and myalgias [7]. Most reports indicate fever and upper respiratory symptoms as the primary initial presentations, consistent with the World Health Organization (WHO)’s list of common symptoms of suspected SARS-CoV-2 infection [8]. In addition, several reports note the extrapulmonary and atypical clinical presentations of COVID-19, not necessarily associated with the typical symptoms of SARS-CoV-2 infection and the related epidemiological risks [4].

However, atypical presentations have appeared since the outbreak of the pandemic in December 2019. They include extrapulmonary involvement such as gastrointestinal symptoms, multiorgan failure (the liver, kidneys, and heart), and neurologic and psychiatric manifestations [9,10,11,12]. It is now clear that patients with COVID-19 may present neuropsychiatric manifestations, including depressive and anxiety symptoms [13], increased risk of suicide [14,15], and insomnia [16]. However, among the psychiatric consequences of COVID-19 infection, delirium appears to be one of the most frequent conditions in emergency settings. Indeed, neuropsychiatric complications may occur early during the infectious disease course and precede typical COVID-19 symptoms [17]. Thus, psychiatrists and infectivologists should manage such manifestations and correlates, carefully monitoring specific populations [18,19] and adopting precautionary measures to contain the spread of COVID-19 [20,21].

Worldwide, scientists are still debating whether neuropsychiatric manifestations originate from SARS-CoV-2’s impact on the brain or result from the psychological distress related to the infection and the pandemic [22]. Thus far, only a few studies have addressed the potential effect of SARS-CoV-2 on mental health. Indeed, the mechanisms underlying the association between COVID-19 and delirium remain unknown, though existing evidence suggests a multifactorial etiology. Considering this, we aimed to review and discuss the mechanisms underlying the neuroinvasive/neurotropic properties of SARS-CoV-2 and the subsequent onset of delirium and neuropsychiatric outcomes.

## 2. Neuroinvasive and Neurotropic Potential of SARS-CoV-2

Human coronaviruses are known to have neuroinvasive potential and neurotropic properties [23]. A direct effect of SARS-CoV-2 on the central nervous system (CNS) was hypothesized after a case of viral encephalitis where SARS-CoV-2 RNA emerged by genome sequencing in the cerebrospinal fluid (CSF) of a patient who experienced a seizure episode [24]. Additionally, autoptic studies identified SARS-CoV-2 RNA in hypothalamic and cortical neurons [25]. However, the mechanisms by which SARS-CoV-2 may extend to CNS and affect brain functioning [24] are scarcely known.

Several studies reported SARS-CoV-2 signals in the brain, particularly endothelial cells and neurons [25,26,27]. However, no infection of glial cells, such as astrocytes and microglia, appeared in vivo. In addition, SARS-CoV-2 infection appeared to be associated with damaged and apoptosed endothelial and neuronal cells [28,29].

### 2.1. The Angiotensin-Converting Enzyme-2 (ACE2) System: A Key to Opening the Blood–Brain Door

SARS-CoV-2 may enter host cells interacting directly with ACE2 receptors, widely present in various tissues, including the brain [29]. Furthermore, SARS-CoV-2 may interact with ACE2 receptors in the capillary endothelium, cause neurovascular abnormalities proximate to infected brain regions and subsequently generate neuronal damage. Varga et al. [30] observed in histological tests the presence of viral elements in the endothelial and inflammatory cells, whose death occurred via apoptosis and pyroptosis. Such findings suggest that SARS-CoV-2 infection facilitates the induction of endotheliitis in several organs, as a direct consequence of the viral infection and the subsequent host inflammatory response.

The endothelitis associated with SARS-CoV-2 infection might impair the systemic microcirculatory function in several vascular beds and generate clinical sequelae in patients with COVID-19. Furthermore, the impaired vascular function may also contribute to cause blood–brain barrier (BBB) destruction that might finally facilitate virus entry into the CNS [31].

Cerebrovascular complications are reported in a considerable number of patients infected by SARS-CoV-2, most presenting ischemic infarcts in small and large arteries, as well as ischemic strokes, and intracranial bleeding [32,33,34].

### 2.2. The Olfactory Nerves and the Lymphatic Drainage System as Alternative Entry Doors to Brain

SARS-CoV-2 might additionally enter the brain via the olfactory nerves located in the nasal cavity and infect neurons that control breathing [35]. Qing et al. [36] speculated that the virus may enter the tears through droplets, cross the nasolacrimal ducts and then the respiratory tract. A recent investigation showed that nearly 89% of patients infected with SARS-CoV-2 requiring intensive care had neurological manifestations and could not breathe spontaneously. Consequently, the patients died from respiratory failure, though it was not demonstrated that neurons controlling breathing were infected [37].

Lechien et al. [38] reported that 85.6% of patients with mild-to-moderate COVID-19 infection presented olfactory dysfunctions. In addition, other reports indicated different degrees of smell impairment [39,40] and postulated the involvement of the olfactory nerves as an entry door to the brain. On the other hand, a magnetic resonance imaging (MRI) case study on a patient with COVID-19 with acute onset of anosmia described a nasal mucosa with no signs of congestion, as well as a normal volume in the bilateral olfactory bulbs [41].

Additionally, Bostanciklioğlu M. [42] speculated that SARS-CoV-2 might enter and spread in the lymphatic drainage system of the brain, regardless of ACE2 receptor system or the olfactory neurons [43].

### 2.3. The Cytokine Storm and Neuroinflammation

Cytokine production is a different pathogenic mechanism associated with a brain infection that remains insufficiently investigated [44,45,46,47]. Indeed, SARS-CoV-2 infection may cause a systemic inflammatory response that results in elevated pro-inflammatory cytokines, chemokines, acute phase proteins, complement, and also in modified of leukocyte profiles in the brain and blood [48]. A large amount of scientific evidence has suggested that a subgroup of patients with severe COVID-19 might present cytokine storm syndrome. Poyiadji et al. [49] hypothesized that a cytokine storm might trigger an acute hemorrhagic-necrotizing encephalopathy resulting from COVID-19, a rare complication that has also been observed in influenza and other viral infections and is postulated to be associated with intracranial cytokines storm.

A common situation triggered by viral infections is also given by hemophagocytic lymphohistiocytosis (HLH), an underrecognized, hyperinflammatory syndrome characterized by a fulminant and fatal hypercytokinaemia with multiorgan failure [50,51]. The primary features of HLH include unremitting fever, cytopenia, hyperferritinaemia, and pulmonary involvement occurring in roughly 50% of affected patients. A cytokine profile resembling HLH is also associated with COVID-19. It is characterized by increased plasma concentrations of interleukin (IL)-2, IL-7, granulocyte colony-stimulating factor (G-CSF), interferon-γ inducible protein 10 (IP-10), monocyte chemoattractant protein 1 (MCP1), macrophage inflammatory protein 1-α (MIP-1α), and tumor necrosis factor-α (TNF-α) [52]. Thus, several proinflammatory factors largely released in patients with COVID-19 might foster neuroinflammation following viral infection. However, more extended investigations are needed to identify all the factors related to the neuroinflammatory process underlying COVID-19.

### 2.4. Hypoxia as a Triggering Factor of Blood–Brain barrier Disruption

Several in vitro and in vivo studies showed that oxygen deprivation might induce BBB disruption, which may trigger neurologic sequelae of COVID-19 [53]. Hypoxia, on the other hand, might also induce paracellular permeability, dysregulation of tight junction protein expression levels, and basement membrane breakdown [54]. Furthermore, hypoxia might increase the non-specific vesicular transport in brain endothelial cells, as shown by increased blood-borne proteins in the brain [55]. Such mechanisms, jointly or separately, facilitate CNS invasion and the diffusion of SARS-CoV-2 throughout the brain, increasing the risks for neuropsychiatric complications in patients with COVID-19 presenting hypoxia.

## 3. SARS-CoV-2 Infection and Psychiatric Outcomes

Psychiatric symptoms, including posttraumatic stress symptoms (PTSS), anxiety and depression, were reported during and after the 2003 SARS-CoV-1 epidemic [56,57]. In particular, Cheng et al. [58] suggested that SARS infection’s direct and indirect effects such as symptom severity, total isolation during the epidemic, and treatment with steroids were likely to contribute to psychiatric complications. In addition, such symptoms were also described in health care workers and the general public during the outbreak of the SARS-CoV-1 epidemic and in the several years after [59,60,61,62,63].

Only a few studies have addressed the potential effect of SARS-CoV-2 on mental health so far. Most studies mainly reported the acute onset of mixed neuropsychiatric conditions in infected patients, characterized by agitated behavior, altered level of consciousness, and disorganized thinking, regardless of psychological or socioeconomic triggering factors. For example, Mao et al. [33] described a case series of patients with COVID-19 with clinical conditions characterized by altered mental status and experiencing an ischemic stroke in about 36% of all hospital admissions. Additionally, Helms et al. [64] reported a case series of patients in an ICU with a high incidence of encephalopathy (85%) and agitation, corticospinal tract signs, and executive dysfunctions. A few patients in this cohort showed enlarged leptomeningeal spaces, while others had bilateral frontotemporal hypoperfusion on the brain MRI. Many patients continued to experience altered cognition even after being discharged [65].

Two additional studies reported on psychiatric symptoms in patients with COVID-19. The first study examined the prevalence of PTSS in clinically stable patients with COVID-19 (96.2% out of 714 hospitalized), who showed significant stress symptoms, as well as poor quality of life and impaired working performance [66]. Under strict lockdown measures, the second study assessed and compared the stress and psychological impact experienced by people with and without psychiatric disorders during the peak of the COVID-19 epidemic. Both psychiatric patients and healthy control subjects were recruited during the 14 days before the restrictions and reported psychiatric symptoms ranging from anxiety and depression to general concerns or stress due to the pandemic. Patients with a psychiatric history reported more severe symptoms when compared with healthy controls and more worries about their physical health, anger/impulsivity, and suicidal ideation. Many patients fulfilled diagnostic criteria for post-traumatic stress disorder (PTSD) [67]; a small percentage suffered from moderate-to-severe insomnia. However, most of the interviewed patients reported no changes or poor/worse physical health status and no increased depression, anxiety and stress [68].

In patients with psychiatric disorders diagnosed before and during COVID-19, a telephone survey by Fernandez-Aranda et al. [69] reported the effects of the first two weeks of confinement in patients with eating disorders. The authors found that almost 38.0% of patients presented worse symptoms and 56.2% experienced anxiety symptoms. Zhou et al. [70] observed worsened symptoms in 20.9% of patients with pre-existing psychiatric disorders without specifying their diagnoses.

In addition, it should be stressed that the pre-existing limited access to mental health services [71] has increased during the COVID-19 pandemic, indirectly contributing to intensify psychiatric problems and exacerbating or increasing substance use disorders [72,73,74,75].

On the whole, the adverse mental health consequences of COVID-19 are predicted but not always precisely measured. Thus, it remains unknown whether psychiatric factors might represent health risk factors for COVID-19.

Taquet et al. [76], in their electronic health record network cohort study, evaluated whether patients diagnosed with COVID-19 showed an increased rate of psychiatric diseases diagnosed after the virus infection. Furthermore, they evaluated whether patients with a history of psychiatric illness in particular were at a higher risk of being diagnosed with COVID-19. Data that emerged from this retrospective cohort study emphasized that survivors of COVID-19 appeared to be at an increased risk for psychiatric sequelae, and that a psychiatric diagnosis might be an independent risk factor for COVID-19. In particular, patients diagnosed with COVID-19 without a psychiatric history showed an increased incidence of a first psychiatric diagnosis in the 14–90 days after the COVID-19 diagnosis, and were at an highest risk of experiencing anxiety disorders, insomnia, and dementia. Specifically, the incidence of the first diagnosis of dementia was 1–6% in people >65 years in the 14–90 days after COVID-19 diagnosis—though, to a smaller extent, similar findings were observed when patients with a previous psychiatric history presented relapses and/or new diagnoses.

In a large group of patients admitted to an academic hospital for suspected COVID-19 pneumonia [77], 11% developed delirium during hospitalization. They were older, had more neuropsychiatric comorbidities and worse respiratory exchanges at baseline. By multivariate models, delirium was independently and positively associated with age, use of antipsychotic drugs, serum urea and lactate-dehydrogenase at admission.

Scientists have expressed concerns regarding the possibility that people with a pre-existing mental disorder may have an increased risk of being infected with COVID-19; on this issue, scarce details are available in the literature so far. Moreover, these patients could have a higher risk of presenting with severe symptoms of COVID-19 infection. This increased risk comes alongside their higher mortality, compared to the general population, due to their poor physical health status [78,79,80] and lifestyle behaviors [81], along with the abuse of substances [82,83,84], which further may contribute to the higher mortality rates in this population. Moreover, it has been documented that, due to the presence of disease-specific symptoms, such as poor cognitive performance [85,86,87], delusions, hallucinations or mood symptoms, patients with psychiatric diseases may have more difficulty in adhering to preventive measures (i.e., wearing masks, maintaining and social distancing), thus increasing the risk of COVID-19 infection.

Pisaturo et al. [88] reported that patients with a previous diagnosis of dementia were more vulnerable than matched control patients without dementia and at increased risk of severe COVID-19 and consequent death.

Wang et al. [89] assessed the impact of a recent (within the last year) diagnosis of a mental disorder, including attention deficit hyperactivity disorder (ADHD), bipolar disorder, depression, and schizophrenia, on the risk for COVID-19, related mortality and hospitalization rates. The most substantial impact of COVID-19 appeared in patients diagnosed with depression and schizophrenia. Additionally, women with mental disorders had higher odds of COVID-19 infection than males, with the most significant gender disparity for ADHD. Patients with a recent diagnosis of a mental disorder and COVID-19 had a death rate of 8.5% (vs. 4.7% among patients with COVID-19 with no mental disorders) and a hospitalization rate of 27.4% (vs. 18.6% among patients with COVID-19 without mental disorders). The authors warned to identify and address modifiable vulnerability factors for COVID-19 infection and prevent delays in health care provision in the psychiatric patient population.

Severance et al. [90] reported that coronavirus exposure might be a comorbidity risk factor in individuals with serious mental disorders. The authors described a unique study population composed of patients who had experienced a recent onset of psychotic symptoms and were subsequently diagnosed with a specific psychiatric disease. Moreover, the authors compared coronavirus immunoglobulin G (IgG) levels of the patients with those of healthy non-psychiatric adults to determine whether a correlation between coronavirus exposure and the recent onset of a serious mental illness existed. Severance et al. [90] concluded that SARS-Co are suitable viruses to study the role that infections in adults play in neuropsychiatric disorders. They invite the scientific community to explore the potential links between the timing of coronavirus infections and the subsequent onset of schizophrenia and other disorders with psychotic symptoms.

## 4. Delirium as the Clinical Onset of SARS-CoV-2 Infection

Delirium is a frequent clinical condition with a negative prognostic trend, mainly observed in hospitalized older adults. Usually, most authors refer to “delirium” to describe an acutely disturbed state of mind characterized by restlessness, illusions, and incoherence, defined by other authors as confusion, altered mental status, acute onset of psychotic symptoms, disorientation, decreased level of consciousness, cognitive dysfunction, and encephalopathy. Furthermore, the clinical appearance of impaired consciousness and/or delirium in most cases of SARS-CoV-2 infection may suggest that the virus penetrates the brain and spreads to the neocortex [42].

Generally, delirium is described in patients following surgery and considered as an indicator of brain vulnerability, and a risk factor for the development of subsequent dementia. In addition, delirium appears as a well-recognized complication of respiratory illness, such as pneumonia. Table 1 illustrates the main predisposing and precipitating factors of delirium.

Nearly a third of patients may develop delirium during an ICU admission, and are at an increased risk of dying while hospitalized. Prolonged hospitalization exposes patients to more significant risks of infections and medical complications that, in some cases, may cause death, besides cognitive impairment after discharge [91]. Indeed, pre-existing cognitive impairment might also represent a significant predisposing factor for the onset of delirium [92,93] (Table 1). Pisaturo et al. [88] reported that patients with dementia are at increased risk of severe COVID-19 and consequent death. Dementia is an insidious neurodegenerative condition, characterized by a chronic and progressive cognitive decline of performance in one or more cognitive domains, interfering with independence in everyday activities. Thus, dementia appears to be the leading risk factor for delirium, though the interrelation between delirium and dementia remains poorly understood. Whether SARS-CoV-2 infection appears as a delirium condition also in patients with a mild pre-existing cognitive impairment is still controversial. However, a few studies have reported on the comorbidity of SARS-CoV-2 infection and delirium in patients with concurrent cognitive impairment in adults ≥18 years.

Table 2 illustrates the results of a literature search showing prevalently case reports and case series of patients presenting delirium or delirium-like symptoms as the main outburst of SARS-CoV-2 infection, plus a mild-to-severe cognitive impairment, which pre-existed or emerged during the acute phase or after SAR-CoV-2 infection (Table 2). In addition, most studies reported that the analyzed patients had a neurologic or psychiatric history including dementia, schizophrenia, major depressive disorders with or without psychotic features, and generalized anxiety disorders. In particular, Zhou et al. [94] indicated that the overall category of delirium, dementia, amnestic, and other cognitive disorders appeared to be predictors for developing new further neuropsychiatric events during hospitalization when already present at admission. However, contrasting data emerged on the potential link between COVID-19 infection and delirium in patients with cognitive impairment and without neuropsychiatric history. Indeed, Parker et al. [95], as well as Wittock and Van Den Bossche [96], reported psychotic symptoms emerging after delirium. On the other hand, Abenza-Abildúa et al. [97] and Hosseini et al. [98] highlighted delirium as a complication of COVID-19 in patients without neuropsychiatric history. The studies of Flores-Silva et al. [99] and Zhou et al. [94] included larger cohorts of patients who developed delirium as in-hospital manifestations and had neurological history, mainly Alzheimer’s disease and dementia. Both studies did not report assessment for the severity of cognitive impairment. Instead, in the case-reports illustrated by Parker et al. [95] and Wittock and Van Den Bossche [96], no psychiatric history emerged, and a psychotic disorder followed delirium. In particular, Parker et al. [95] demonstrated that the patient manifested a degree of cognitive impairment (visuospatial/executive reasoning, persistent errors in abstraction, language, and attention) until the time of discharge. In contrast, the patient described by Wittock and Van Den Bossche [96] had a premorbid mild cognitive impairment. In the remaining four case reports [97,98,100,101], all patients showed a mild cognitive impairment after recovery and/or at hospital admission. Finally, all the patients described by Beach et al. [102] and Anmella et al. [103] had a neuropsychiatric history (dementia, schizophrenia, or depressive disorders). In particular, patients included in the study of Beach et al. [102] had a premorbid cognitive impairment. Differently, in the study of Anmella et al. [103], only one patient showed a moderate intellectual impairment.

SARS-CoV-2 infection might cause delirium in a significant percentage of patients in the acute stage and patients with a pre-existing mental disorder might represent a population with increased risks associated to being infected with COVID-19. Conversely, there remains little known about patients infected with SARS-CoV-2 presenting delirium without psychiatric history.

In this regard, Taquet et al. [76] state that patients with COVID-19 infection without a psychiatric history showed an increased incidence of a first psychiatric diagnosis in the following 14–90 days. On the contrary, Mazza et al. [104] reported that cognitive impairment (dysfunctions in attention and information processing) was strictly related to the presence of depressive symptoms in the three months following the viral infection and systemic inflammation. Furthermore, the authors found no significant difference in cognitive performance tests between patients with and without a previous psychiatric history.

It remains challenging to determine whether the psychological response to COVID-19 may contribute to patients’ neuropsychiatric manifestations. Thus, several factors might be identified as trigger events influencing the outcome of COVID-19.

Patients with delirium are also time consuming for clinicians, and their functioning is often poor, with high additional costs for the health care system [92,93]. Such patients are likely to require a more extended presence of hospital staff and the use of life-support resources, mainly due to the frequent in-hospital complications. However, delirium may sometimes result in a transfer of patients to long-term facilities, shortly afterward interrupted for hospital readmission for medical complications. Such a vicious cycle burdens the healthcare system, as it is occurring during the perduring COVID-19 pandemic.

In the acute phase of COVID-19 in older patients, delirium might be assessed and managed belatedly or insufficiently. The reason is that physicians prioritize the diagnosis and treatment of COVID-19 and only later involve mental specialists.

The mechanisms underlying the association between COVID-19 and delirium are still unknown. However, the existing evidence suggests a multifactorial etiology and concurrent factors as direct CNS invasion, cerebrovascular involvement, and more indirectly through hypoxia, high fever, dehydration, inflammation (cytokine storm), medications, or metabolic derangements.

The current assessment of COVID-19, which includes several national guidelines, does not contemplate delirium or mental status changes as symptoms, especially regarding older adults. Because of this, the risk of not considering delirium in the screening criteria for patients with COVID-19 is high. The phenomenon appears relevant in care homes, where evidence emerges of high mortality rates associated with delirium, as well as the risk for subsequent long-term cognitive and functional decline [105].

Delirium rates differed substantially depending on the study population and diagnostic settings. The prevalence of delirium was high in patients admitted to an ICU, ranging from 65% to 79.5% [106,107]. In studies that stratified groups of patients based on COVID-19 severity, higher rates of delirium were reported in those with severe respiratory disease. The delirium condition presented variously, such as disorder of consciousness (7.2% in patients without severe respiratory disease vs. 38.9% in patients with severe respiratory disease), acute confusional syndrome (3.9% in patients without severe respiratory disease vs. 14.9% in patients with severe respiratory disease), confusion (0% in patients without severe respiratory disease vs. 18.5% in patients with severe respiratory disease), and impaired consciousness (2.4% in patients without severe respiratory disease vs. 14.8% in patients with severe respiratory disease). Similarly, studies of older adults found that significant percentages of patients (29–40%) experienced delirium while hospitalized due to COVID-19 infection, often associated with comorbidities such as increasing age and frailty [95]. Furthermore, individuals presenting with neurological symptoms and COVID-19 were more likely to have delirium/altered mental status than those with neurological symptoms who did not have COVID-19 (26.8% in patients with neurological symptoms and COVID-19 vs. 7.7% in patients with sole neurological symptoms) [108].

Marengoni et al. [108] reported that, in a total sample of 91 patients infected with COVID-19 (aged ≥ 70 years), 25 patients had a diagnosis of delirium (27.5%), and 39 patients died during hospitalization, meaning that the risk of in-hospital mortality is four times greater than in patients without delirium. In addition, Jäckel et al. [109] highlighted that patients with delirium died due to COVID-19 pneumonia, sepsis and related severe complications in most cases.

Maiese et al. [110] indicated that in patients with SARS-CoV-2 infection, the post-mortem analyses demonstrated mainly hypoxic changes as the most frequently reported alteration of brain tissue, followed by ischemic and hemorrhagic lesions plus reactive astrogliosis and microgliosis. Since these findings are not specific to SARS-CoV-2 infection, the authors hypothesized a more likely association with systemic inflammation and coagulopathy caused by COVID-19. Patients hospitalized with COVID-19 face additional challenges. The current hospital management of COVID-19 involves isolation, limitation of family visits, and even limited physical contact with hospital staff. Moreover, hospital staff’s use of personal protective equipment may be depersonalizing and frightening to older patients, particularly those with dementia or a cognitive impairment. Medical tests are often performed late at night to ensure adequate time for equipment sterilization. The hospital routine may often disrupt the patient’s sleep and cause disorientation to those who appear to be more vulnerable.

Overall, the current medical approach results in almost total social isolation, with increased use of both physical and chemical restraints to manage fear, agitation, and wandering. Such measures may increase the risk of developing delirium. Moreover, this type of approach may exacerbate and extend the duration of the neuropsychopathological condition, leading to worse outcomes potentially liable to accelerate mortality.

Currently, whether the high rate of delirium is associated explicitly with SARS-CoV-2 infection or rather a common complication of the acute respiratory distress syndrome (ARDS) remains controversial. Jäckel et al. [109] reported that delirium duration and severity in patients with ARDS caused by either SARS-CoV-2 or influenza A and B viruses tended to be higher in patients with SARS-CoV-2. Therefore, the authors hypothesized that delirium observed in patients with COVID-19 was considered a complication of ARDS rather than a direct SARS-CoV-2 specific invasion of the brain. Instead, Hosseini et al. [98] reported on two patients with COVID-19 presenting an acute onset of altered mental status and subsequent delirium, normal respiration and metabolic balance, who later developed symptoms of neuroinflammation. Thus, the authors hypothesized that the clinical condition observed in both patients was probably associated with a neuroinfection or an autoimmune encephalopathy. Furthermore, Cuperlovic-Culf et al. [111] hypothesized a relationship between delirium and SARS-CoV-2 infection via a possible binding of SARS-CoV-2 spike protein to monoamine oxidase B (MAO-B) enzymes, which influences the enzyme activity and possibly leads to many of the observed neurological and platelet-based complications of SARS-CoV-2 infection.

## 5. Conclusions

Psychiatric manifestations and complications related to the SARS-CoV-2 pandemic are still under investigation and not entirely elucidated.

Research evaluating the direct and indirect consequences of the virus infection on mental health is required to improve treatment, mental health care planning, and prevention of long-term sequelae during this pandemic and any other possible pandemics in the future [3]. In addition, this research area needs to be expanded, since the dynamics and the presentation spectrum of the COVID-19 may vary enormously in the general and psychiatric population.

Several studies highlighted that a new mental disease episode might represent a clinical manifestation of COVID-19 sequelae, especially for patients with a psychiatric history. On the other hand, other studies also emphasized that delirium may be the sole clinical manifestation of SARS-CoV-2 infection associated with the spread of the virus throughout the CNS in patients without a psychiatric history.

Therefore, clinicians should consider the possibility that COVID-19 may be directly responsible for acute psychiatric complications, even in the absence of other systemic symptoms. In addition, as already reported, mainly for patients with dementia, a limited number of studies stressed that a mild cognitive impairment before SARS-CoV-2 infection might also be a possible predisposing factor to COVID-19.

The findings in the literature should direct research to establish the role of viral neurotropism, host immune responses, and genetic factors liable to complications to achieve more suitable treatments.

## Figures and Tables

**Table 1:** Predisposing and precipitating factors for delirium.

**Predisposing Factors**
Older age;
Dementia or pre-existing cognitive impairment;
Previous delirium episodes;
Functional impairment;
Sensory impairment (e.g., vision and/or auditory disabilities);
Presence of comorbid medical illnesses;
Pre-existing neuropsychiatric disorders (e.g., depression, alcohol use disorder).
**Precipitating Factors**
Polypharmacotherapy (e.g., concomitant use of sedative-hypnotic drugs, diuretics, anticoagulants, antibiotics);
Use of physical restraints;
Use of bladder catheter;
Infections;
Major surgery;
Trauma or urgent admission to hospital;
Coma;
Metabolic abnormalities (e.g., abnormal serum sodium, glucose or potassium concentrations, hypoxemia, metabolic acidosis).

**Table 2:** Neuropsychiatric history, type of symptoms at admission or during hospitalization, and cognitive impairment reported by studies including patients with COVID-19 with delirium.

Authors	Study Type	Number, Gender and Age of Patients (%)	Patients with or without Neuropsychiatric History (*n*, %)	Symptoms at Admission/during Hospitalization (*n*, %)	Cognitive Impairment
**Flores-Silva et al., 2021 [99]**	Prospective, cross-sectional, observational	375 F (35%), 697 M (65%), 53.2 ± 13 years	71 (6.6%)	Delirium (*n* = 140, 13.1%)	N/A
**Parker et al., 2021 [95]**	Longitudinal case report	M, 57 years	None	Acute psychotic symptoms	yes
**Zhou et al., 2021 [94]**	Observational cohort	509 F (46.6%), 582 M (53.35), 57.17 ± 9.23 years	34 Dementia (3.1%) 20 Alzheimer’s disease (1.8%)	The overall category of delirium, dementia, amnestic and other cognitive disorders *	yes
**Wittock and Van Den Bossche, 2020 [96]**	Case report	F, 88 years	None	Delirium with delusional disorder	Premorbid mild cognitive impairment
**Beach et al., 2020 [102]**	Case series	Total patients: 4 —M, 76 years —M, 70 years —M, 68 years —F, 87 years	—Major neurocognitive disorder with episodic agitation and psychotic features —Dementia with Levy bodies —Schizophrenia —Major depressive disorder with psychotic features	—Respiratory symptoms followed by delirium —Only fever, followed by alterations of speech, spontaneous bilateral myoclonus, catatonic behavior —Inability to follow commands and incomprehensible speech, disorientation and impulsiveness for several days —Initial anxiety and dysphoria, then agitation, disorientation, concern about physical symptoms, mumbling, slurred speech	All with premorbid cognitive decline
**Gillett and Jordan, 2020 [100]**	Case report	M, 37 years	No personal psychiatric history, positive family psychiatric history	Confusion, bizarre behavior, visual and auditory hallucinations, self-harm behavior (suicide attempt).	Mild, after recovery
**Abenza-Abildúa et al., 2020 [97]**	Case report	F, 56 years	None	Delirium after respiratory symptoms, resolved within 72 h	N/A
**Palomar-Ciria et al., 2020 [101]**	Case report	M, 65 years	Stable schizophrenia (>20 years)	Agitated delirium	Mild, during confusional state
**Anmella et al., 2020 [103]**	Case series	Total patients: 4 —M 68 years —M 53 years —M 61 years —F 68 years	—Previous depressive episode —Unspecified psychosis —Delusional disorder —Depressive and generalized anxiety disorder	—Confusion, agitated behavior —Behavioral disturbances —COVID pneumonia —Insomnia, worsening of anxiety and mood liability	—N/A —Moderate intellectual disability —N/A —N/A
**Hosseini et al., 2020 [98]**	Letter to the Editor	Total patients: 2 —M 46 years —F 79 years	—None —None	—Ankle clonus followed by status epilepticus after 2 days —Confusion and verbal communication difficulties followed by impaired orientation, attention and memory.	—After a week, mild impairments (verbal fluency, linguistic abstraction, phrase repetition, and delayed recall memory); —After recovery, persisted impaired verbal fluency, repetition, abstraction, and delayed recall memory

* All predictors for the development of new neuropsychiatric events during hospitalization when present at admission. N/A = Not applicable.

## Data Availability

Data presented in this review are available in the tables.
